# Distinct genetic alterations occur in ovarian tumor cells selected for combined resistance to carboplatin and docetaxel

**DOI:** 10.1186/1757-2215-5-40

**Published:** 2012-11-30

**Authors:** Stephen R Armstrong, Rashmi Narendrula, Baoqing Guo, Amadeo M Parissenti, Katherine L McCallum, Stephanie Cull, Carita Lannér

**Affiliations:** 1 Dept. of Biology, Laurentian University, Sudbury, ON, P3E2C6, Canada; 2 Sudbury Regional Cancer Center, Cancer Research Laboratory, Sudbury, ON, P3E5JI, Canada; 3 Division of Medical Sciences, Northern Ontario School of Medicine, Laurentian University campus, Sudbury, ON, P3E2C6, Canada; 4 Northern Ontario School of Medicine, 935 Ramsey Lake Road, Sudbury, ON, P3E2C6, Canada

**Keywords:** Ovarian cancer, Multidrug resistance, Carboplatin, Docetaxel, Microarray analysis, A2780 cell line

## Abstract

**Background:**

Current protocols for the treatment of ovarian cancer include combination chemotherapy with a platinating agent and a taxane. However, many patients experience relapse of their cancer and the development of drug resistance is not uncommon, making successful second line therapy difficult to achieve. The objective of this study was to develop and characterize a cell line resistant to both carboplatin and docetaxel (dual drug resistant ovarian cell line) and to compare this cell line to cells resistant to either carboplatin or docetaxel.

**Methods:**

The A2780 epithelial endometrioid ovarian cancer cell line was used to select for isogenic carboplatin, docetaxel and dual drug resistant cell lines. A selection method of gradually increasing drug doses was implemented to avoid clonal selection. Resistance was confirmed using a clonogenic assay. Changes in gene expression associated with the development of drug resistance were determined by microarray analysis. Changes in the expression of selected genes were validated by Quantitative Real-Time Polymerase Chain Reaction (QPCR) and immunoblotting.

**Results:**

Three isogenic cell lines were developed and resistance to each drug or the combination of drugs was confirmed. Development of resistance was accompanied by a reduced growth rate. The microarray and QPCR analyses showed that unique changes in gene expression occurred in the dual drug resistant cell line and that genes known to be involved in resistance could be identified in all cell lines.

**Conclusions:**

Ovarian tumor cells can acquire resistance to both carboplatin and docetaxel when selected in the presence of both agents. Distinct changes in gene expression occur in the dual resistant cell line indicating that dual resistance is not a simple combination of the changes observed in cell lines exhibiting single agent resistance.

## Background

Ovarian cancer remains the most lethal gynecological cancer, with a 5-year mortality rate greater than 50% [1]. The high mortality rate from ovarian cancer is partly due to lack of effective screening and diagnosis methods and another significant factor is the development of resistance to chemotherapeutic treatment regimens [2,3]. The advanced stage of most tumours at diagnosis has led to cytoreductive surgery with subsequent chemotherapy as the current standard of treatment for ovarian cancer [4,5]. Despite the high rates of initial response, more than half of all patients will experience recurrent disease and eventually fail to respond to chemotherapy [6]. Failure of chemotherapy in recurrent ovarian cancer is usually due to the development of resistance to the two main classes of chemotherapy agents used to treat ovarian cancer, platinating agents and taxanes, and combined resistance to both agents may occur [7-9].

Mechanisms underlying the development of resistance to platinating agents, especially cisplatin, have been well characterized and include repair of DNA lesions, translesional DNA synthesis, altered cellular transport of the drug, increased antioxidant production, and reduction of apoptosis [10-12]. Altered gene expression affecting cellular transport, DNA repair, apoptosis, and cell-cell adhesion are mechanisms of platinum resistance that have been observed in patient samples [13,14]. In the treatment of ovarian cancer, taxanes were originally introduced as an alternative to cisplatin and to overcome cisplatin resistance [15,16]. The development of resistance to taxanes has been equally well studied and genetically characterized. Typical mechanisms of paclitaxel resistance involve alterations in drug transport, e.g. changes in P-glycoprotein expression, altered expression of or mutations in microtubule protein genes, expression of taxane metabolizing proteins, and altered cell signaling resulting in reduced apoptosis [17-20]. Although clinical evidence indicating a role for some of these factors in patient response to taxane treatment of cancer, e.g. altered expression of Class III β-tubulin, reduced apoptosis conferred by survivin expression and metabolism of taxanes by cytochrome P450 proteins, clinical evidence for many mechanisms established in preclinical models is variable [21-23][24,25].

The difference in mode of action and mechanisms of resistance between platinating agents and taxanes is taken advantage of in dual agent chemotherapy of advanced ovarian cancer, to achieve significantly increased efficacy and progression free survival (PFS) of patients. The most common combination therapy is carboplatin together with paclitaxel, although the taxane docetaxel has also been used with similar efficacy [26-28]. Notwithstanding the success of dual agent therapy, relapse of the cancer and development of resistance occurs in the majority of cases [4,26,29,30]. Chemoresistance arising from combined platinating agent and taxane therapy is more difficult to overcome than single agent resistance [31-33]. Currently, it is not known if mechanisms of resistance to dual agent chemotherapy are a combination of single agent resistance responses or if novel mechanisms arise as a result of combination therapy. Moreover, it is difficult to overcome dual drug resistance, even with drugs that have completely different modes of action and targets [34-36]. This may indicate that novel and different mechanisms of resistance arise from combined platinating agent/taxane chemotherapy. In this study, carboplatin was selected as the platinating agent based on its’ common clinical use [7,37,38]. Docetaxel was chosen as the taxane agent based on the potentially favorable toxicity profile [27], especially when combined with pegfilgrastim to prevent neutropenia [39], and increasing use for cancers like breast cancer. Furthermore, docetaxel has been shown to have activity against paclitaxel resistance in patients [40].

To investigate if the development of dual agent resistance invokes different mechanisms or is a combination of the mechanisms of resistance that arise upon exposure to single agents, we have developed a set of isogenic ovarian cancer cell lines resistant to either carboplatin, docetaxel or a combination of carboplatin and docetaxel. Changes in gene expression associated with the specified drug resistance in each cell line were analyzed using microarray analysis. Comparison between the three resistant cell lines permitted identification of shared and different changes in gene expression among the cell lines. This analysis showed that the establishment of carboplatin and docetaxel resistance does not share many changes in gene expression and that dual agent resistance appears to develop from mostly unique changes in gene expression, different from both carboplatin and docetaxel resistance in the set of isogenic cell lines studied.

## Methods

### Cell lines and culture

The human ovarian carcinoma cell line A2780 was purchased from the European Collection of Cell Cultures (ECACC, Salisbury, UK) and maintained in RPMI-1640 medium with 2mM Glutamine, which contained 10% fetal bovine serum (FBS), and 1% Penicillin (10,000 U/ml)/Streptomycin(10,000 μg/ml) solution (HyClone, South Logan, Utah, US). The A2780 ovarian cancer line is likely of the endometrioid subtype (Dr. Michael Anglesio, data pending publication). The drug resistance of the carboplatin resistant cell line A2780CBN was maintained by adding 2.22 × 10^-5^ M carboplatin in complete medium (RPMI-1640 with 10% FBS and 1% Penicillin 10,000 U/ml/Streptomycin 10,000 μg/ml) once every week, the docetaxel resistant cell line A2780DXL was maintained by adding 4.05 × 10^-7^ M docetaxel in complete medium bi-weekly and resistance of the carboplatin/docetaxel dual resistant cell line A2780CBNDXL was maintained by treating with 6.07 × 10^-6^ M carboplatin and 6.07 × 10^-9^ M docetaxel in complete medium bi-weekly.

### Cell viability assay

#### Clonogenic assay for drug sensitivity

Cells were assayed for sensitivity to carboplatin, docetaxel, and combined carboplatin/docetaxel using a clonogenic assay that quantifies the number of colonies generated from viable cells [41].

#### Determination of IC_50_

The number of colonies growing was recorded by taking photomicrographs of five random fields (100X magnification) per drug concentration and counting the colonies in each field. The average for each drug concentration was normalized to the average of the drug free control to generate a survival fraction. The software program Graph Pad Prism (Graph Pad Software Inc, La Jolla, CA) was used to plot a survival curve using the function “log [inhibitor] vs. normalized response with variable slope” to calculate the IC_50_. Statistical analysis to determine if the IC_50_ of each resistant line was significantly different from the co-cultured parental control was calculated using Student’s t-test with two-tailed distribution and unequal variance. A p-value ≤0.05 indicated that a significant difference existed between the two sets of data (n = 3).

### Cell line selection

Cell line selection was performed as described in a previous study by Guo et al. [42]. Briefly, selection began in a dose 1000-fold below the IC_50_ of the parent line and doses were increased 3.00-fold, 1.50-fold or 1.25-fold, depending on the ability of the cells to continue proliferating. For the A2780CBNDXL cell line, the concentrations of carboplatin and docetaxel were raised together by the same factor each time. A co-cultured control was developed for each resistant line, as described, to control for changes in gene expression due to continuous culture.

### Cell line growth rate analysis

Cells were plated at a density of 2.0 × 10^5^ cells per 9.60 cm^2^ in six well plates. Three wells were counted for each day of a four day growth curve analysis using a Vi-cell XR cell viability analyzer (Beckman Coulter, Inc., Mississauga, ON). Three biological replicates of the growth curve experiment were performed. Averages of viable cell numbers were plotted for each day and student’s t-test (unpaired, two tail, variable variance) was applied to determine if the average cell number per day was significantly different or not from the parental line. Population doubling time was calculated using the formula *G* = *t* * *log*(2)/(log(*N*_*t*_) − log(*N*_0_)), where G = generation or doubling time, t = time period (hr.), N_t_ = number of cells at time t, N_0_ = initial number of cells.

### RNA isolation and quality analysis

Total RNA from each cell line was prepared using RNeasy Mini Kit (50) # 74104 from Qiagen Inc. (Toronto, ON) according to the manufacturer’s instructions. RNAase OUT (cat. 10777019,Invitrogen/Life Technologies ) was added to prevent RNA degradation. Integrity of total RNA samples was assessed using capillary electrophoresis on an Agilent 2100 Bioanalyzer. RNA samples with RIN values of 8.0 or higher were considered intact and appropriate to use for microarray analysis.

### Microarray analysis

Changes in gene expression between A2780 parental cells and the derived drug resistant cell lines were observed using Agilent 4 × 44 whole human genome arrays (Product #G4112F; Agilent Technologies, Mississauga, ON). A 500 ng aliquot of total RNA, isolated with a Qiagen RNeasy mini kit (product # 74104, Qiagen Inc.) was used for each sample. The RNA was labeled with Cy3 or Cy5 using and Agilent Quick Amp Labeling kit (Product # 5190–0444). Hybridization was performed as per the manufacturer’s protocol. Experiments were repeated using multiple batches of labeled RNA, with both forward and reverse-labeling to account for dye bias, for a total of 4 (A2780CBN and A2780CBNDXL) or 8 (A2780DXL) two-color arrays. The microarrays were scanned, and feature extraction and background intensity corrections were performed with Agilent software (v.10.7.3.1). Using a 3-way ANOVA, Partek Genomics suite (St. Louis, MO) was used to generate a list of genes significantly over- or under-expressed with false discovery rates of 0.01 and 0.05, with a cut-off value of ± 2-fold change in gene expression. The microarray data was deposited in the NCBI Gene Expression Omnibus (GEO) database in accordance with MIAME standards (GSE39337). This list was further refined in our analysis by only including genes which had p≥0.05 for the comparisons between replicate arrays and reverse labeled samples. The refined gene lists were imported into Microsoft Office Excel 2003 to perform a three-way column comparison to identify genes that were unique to each cell line, shared between two lines or shared between all three of the resistant lines. Partek Genomics Suite was also used to perform principal component analysis (PCA) and hierarchical clustering analysis of the data.

### Quantitative real time PCR (QPCR)

Three independent RNA isolations were prepared from each cell line using Qiagen RNeasy isolation kits. Reverse transcription of cDNA was performed using the Superscript First-Strand synthesis system for QPCR from Invitrogen Canada Inc. (Burlington, ON). Sybrgreen and Taq polymerase reagents for the QPCR reactions were purchased as a GoTaq QPCR Master Mix from Promega Corporation (Madison, WI). QPCR reactions were carried out on a BioRad Dyad Disciple Peltier Thermal Cycler using a Chromo4 Real-time PCR Detector. QPCR primers were designed to specifically amplify coding transcripts (Additional file 1: Table S1). The S28 ribosomal RNA gene was chosen as a housekeeping gene as transcript levels did not vary between the resistant and parent A2780 lines.

### Immunoblotting

Total protein lysates were resolved by SDS-PAGE and transferred to nitrocellulose membranes. Antibodies for ABCB1 (cat. sc-73354), GCLC (cat. sc-100747), FLRT3 (cat. sc-82156), CDH11 (cat. sc-52352), CYP1B1 (cat. sc-32882), ANXA1 (cat. sc-12740), GAPDH (cat.sc-47724) and GSTO1 (cat. sc-130318) were purchased from Santa Cruz Biotechnology, Inc. (Santa Cruz, CA). The antibody for MT2A (cat. H00004502-M01) was purchased from Abnova Corporation (Cedarlane Laboratories Ltd., Burlington, ON). Antibodies against AKR1C3 (Clone NP6.G6.A6) and γ-tubulin (cat. T5192) were acquired from Sigma-Aldrich Canada, Ltd. (Oakville, ON). Binding of antibodies was detected using Luminol enhanced chemoluminescence (ECL) reagents from Santa Cruz Biotechnology, Inc. and images were recorded and analyzed using an Alpha-Innotech gel documentation system with Alpha-Ease software system (Cell BioSciences, Inc., Toronto, ON) or by exposing film.

### Statistical analysis of changes in gene expression

For the microarray data, significant differences in fold change expression between co-cultured controls and resistant lines was determined using the Partek Genomics Suite program (Partek Inc.,St. Louis, MO). Significant differences between log Ct values for the parent and resistant lines, normalized to log Ct values for the S28 transcript, were determined using Student’s t-test. To determine fold change for the qPCR data, the average relative quantity of gene expression for each gene was determined using MJ Opticon Monitor Analysis Software v. 3.1 (BioRad Laboratories, Inc., Mississauga, ON)). Following normalization to the S28 housekeeping gene, the fold changes were determined from the ratio between parental and resistant lines. ANOVA followed by Tukey’s post hoc test was calculated for qPCR and immunoblot log fold change data in the Graphpad Prism v. 5.02 (GraphPad Software, Inc., San Diego, CA).

## Results

### Generation of carboplatin, docetaxel, and carboplatin/docetaxel resistant cell lines

The original A2780 parent line had an IC_50_ for carboplatin of 2.12 × 10^-6^ M. Therefore, selection for the carboplatin resistant line began at 1.00 × 10^-9^ M carboplatin, a dose in the 1000 fold range below the IC_50_ of the parent line, and continued until a maximally tolerated dose (MTD) of 2.22 × 10^-5^ M was reached. A maximally tolerated dose was considered to be achieved when cell viability dropped below 30% at the next higher dose. Clonogenic assays performed on the A2780CBN cell line at this point revealed an IC_50_ of 7.77 x 10^-5^ M to carboplatin, while the A2780 co-cultured parental control (A2780CC) had an IC_50_ of 5.73 × 10^-6^ M carboplatin. The IC_50_ values for the A2780CBN and A2780CC were statistically different by Student’s t-test (n=3, p=0.004). The ratio between the resistant and co-cultured control IC_50_ values demonstrated about a 13-fold increase in the IC_50_ of the resistant A2780CBN line (Table 1). The docetaxel selection was performed in a similar fashion, beginning with determination of IC_50_ of the original parent line as 8.82 × 10^-10^ M DXL. Selection in docetaxel started in 1 × 10^-13^ M, and ended with a MTD of 4.05 × 10^-7^ M. The IC_50_ and fold-resistance of the A2780DXL line and corresponding A2780CC are shown in Table 1.

**Table 1:** **Resistance values determined by clonogenic assay expressed as IC**_**50**_**for each cell line and drug**(**s**)

**Resistant line**	**IC**_**50**_**of resistant line to either CBN and**/**or DXL**	**IC**_**50**_**of co**-**cultured control to either CBN and**/**or DXL**	**Fold resistance**. **Ratio of IC**_**50**_**of resistant line**/ **co**-**cultured control**
A2780CBN	7.77 x 10^-5^ M CBN	5.73 x 10^-6^ M CBN	13.56
	3.62 x 10^-10^ M DXL	5.76 x 10^-10^ M DXL	0.63
A2780DXL	3.61 x 10^-7^ M DXL	8.91 x 10^-11^ M DXL	4051.63
	2.20 x 10^-6^ M CBN	6.75 x 10^-6^ M CBN	0.33
A2780CBNDXL	8.02 x 10^-6^ M CBN	6.37 x 10^-7^ M CBN	12.59
	8.02 x 10^-9^ M DXL	6.37 x 10^-10^ M DXL	
A2780CBNDXL	2.52 x 10^-5^ M CBN	2.50 x 10^-6^ M CBN	10.08
A2780CBNDXL	1.47 x 10^-8^ M DXL	1.84 x 10^-9^ M DXL	7.99

#### Selection of the A2780CBNDXL dual resistant cell line

**Figure 1:**
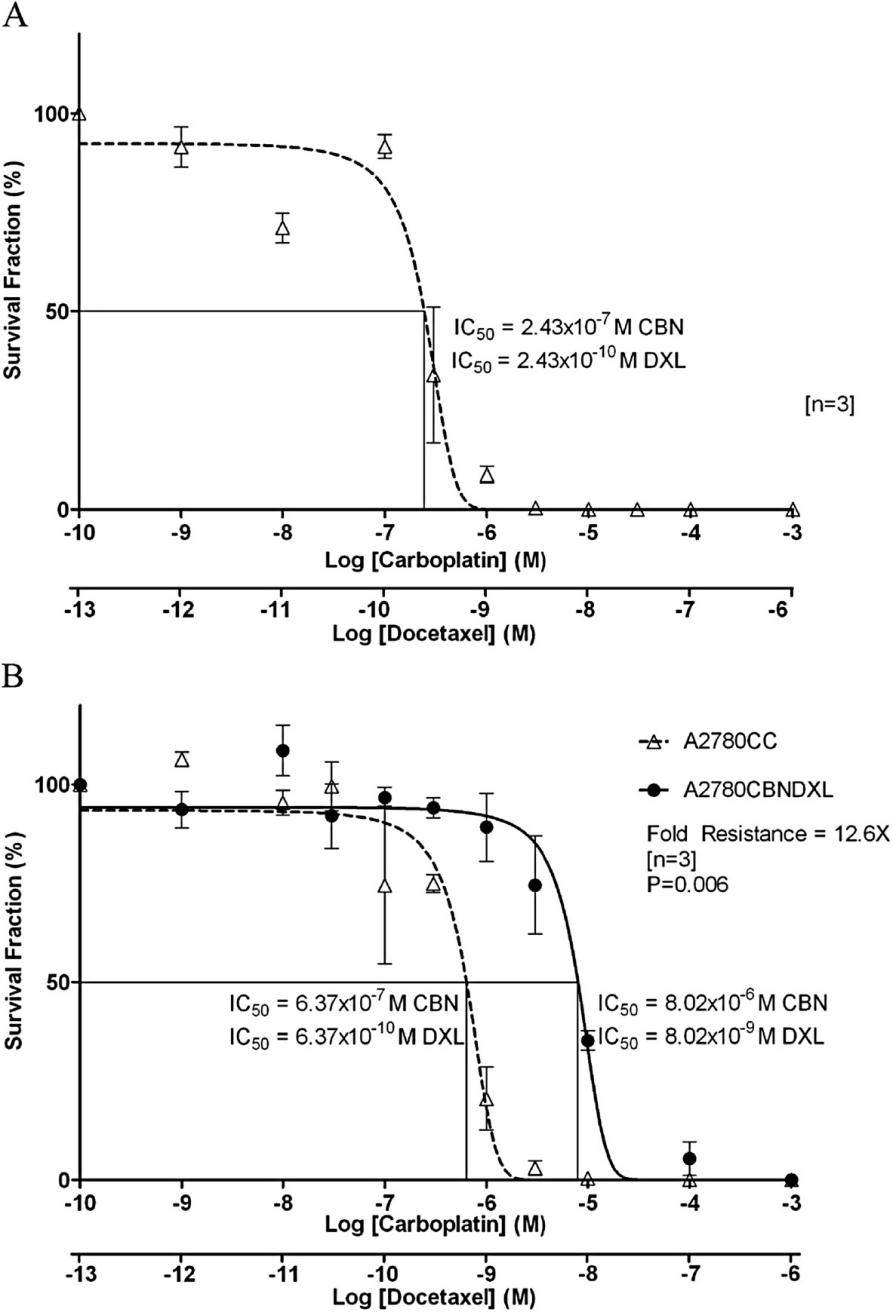
**Response of A2780**, **A2780CBNDXL and A2780CC to combined carboplatin and docetaxel.** Dose–response curves showing the survival fraction (%) of (**A**) A2780 parent or (**B**) A2780CBNDXL and A2780CC cell colonies exposed to increasing concentrations of the two drugs expressed as the log of carboplatin and docetaxel concentration in molarity (M).

Based on the drug concentrations used to begin selection for the A2780CBN and A2780DXL lines, a combination of 1 × 10^-9^ M carboplatin and 1 × 10^-13^ M docetaxel was used to begin selection for the dual resistant line. When exposed to combined carboplatin and docetaxel, the IC_50_ values of the A2780 parent line were 2.43 × 10^-7^ M for carboplatin, and 2.43 × 10^-10^ M for docetaxel (Figure 1A). This was lower than the IC_50_ of the parent cells to carboplatin alone by a factor of 8 fold and lower than the IC_50_ of the parent cells to docetaxel alone by a factor of 4 fold, a result that demonstrates the increased efficacy of combining the two drugs.

Selection was carried out until a maximally tolerated dose of 6.07 × 10^-6^ M carboplatin and 6.07 × 10^-9^ M docetaxel was achieved. However, the IC_50_ of the A2780CBNDXL cells was 8.02 x 10^-6^ M carboplatin, and 8.02 × 10^-9^ M docetaxel, almost 13 fold higher than the IC_50_ for the A2780CC cells at a similar passage number (Figure 1B and Table 1), and 33 fold higher than the IC_50_ of the original A2780 parent cells (Figure 1A).

### Proof of dual resistance in the A2780CBNDXL cell line

**Figure 2:**
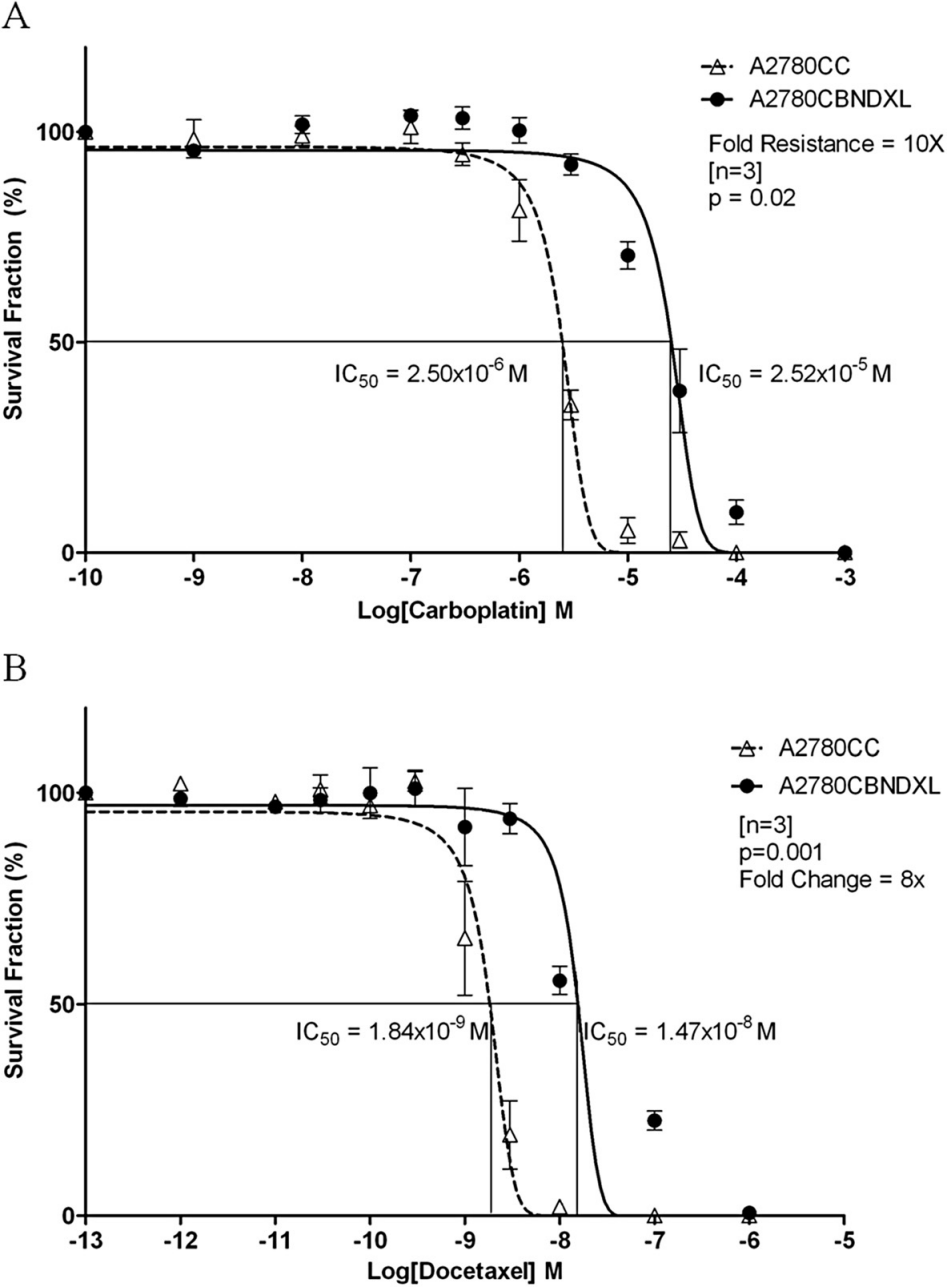
**Resistance of the A2780CBNDXL cell line to each drug alone.** Dose–response curves showing the survival fraction (%) of A2780CBNDXL and A2780CC colonies exposed to increasing concentrations of (**A**) carboplatin or (**B**) docetaxel, expressed as the log of concentration in molarity (M).

To establish that the A2780CBNDXL line was genuinely resistant to both carboplatin and to docetaxel, the cell line was exposed to each drug separately in two clonogenic assays. The results, shown in Figure 2 and Table 1, demonstrate that the A2780CBNDXL line is resistant to both carboplatin and docetaxel and is, therefore, a dual drug resistant cell line.

### Lack of cross resistance in the A2780CBN and A2780DXL lines

**Figure 3:**
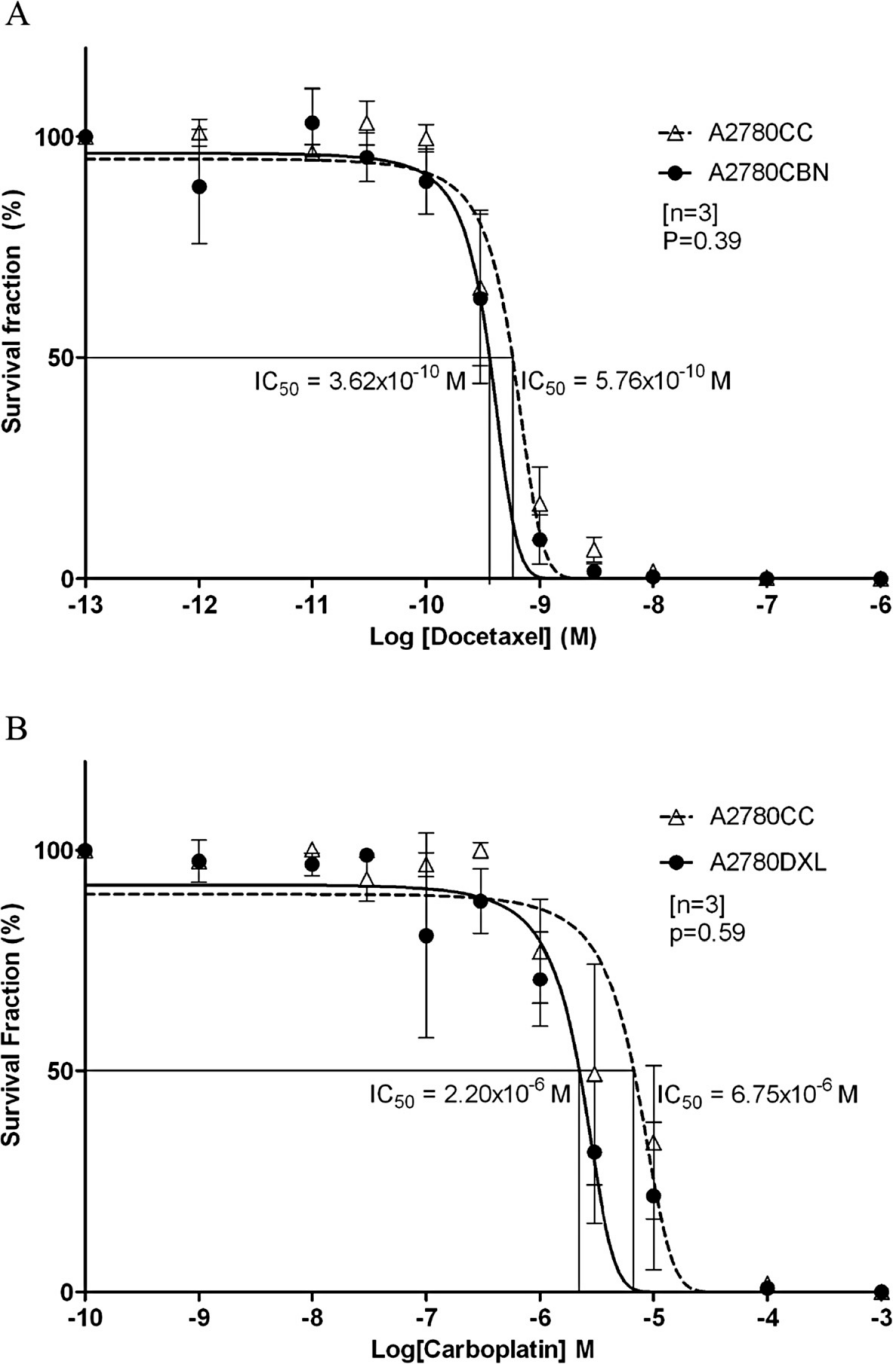
**Lack of cross**-**resistance in the A2780CBN and A2780DXL cell lines.A**. Dose response curve showing the survival fraction (%) of A2780CBN and A2780CC colonies exposed to increasing doses of docetaxel. **B**. Dose response curve showing the survival fraction (%) of A2780DXL and A2780CC colonies exposed to increasing doses of carboplatin.

The A2780CBN cell line was exposed to varying concentrations of docetaxel along with the co-cultured parental control and plated in a clonogenic assay. The A2780CBN line had an IC_50_ of 3.62 × 10^-10^ M in docetaxel while the A2780CC displayed an IC_50_ of 5.76 × 10^-10^ M (Figure 3A and Table 1). The responses of the two cell lines to docetaxel were compared for statistical significance using Student’s t-test and were not significantly different (p=0.39, n=3), indicating that the A2780CBN cell line was not cross resistant to docetaxel.

A clonogenic assay was also carried on the A2780DXL cell line to test for cross resistance to carboplatin. Plotting of the data generated an IC_50_ value of 2.20 × 10^-6^ M carboplatin for the A2780DXL cell line and 6.75 × 10^-6^ M for the A2780CC (Figure 3B and Table 1). There was no significant difference between the IC_50_ values (p=0.59, n=3) for the A2780DXL and A2780CC cell lines, establishing a lack of cross resistance to carboplatin in the A2780DXL line.

### Proliferation of the resistant and parental cell lines

**Figure 4:**
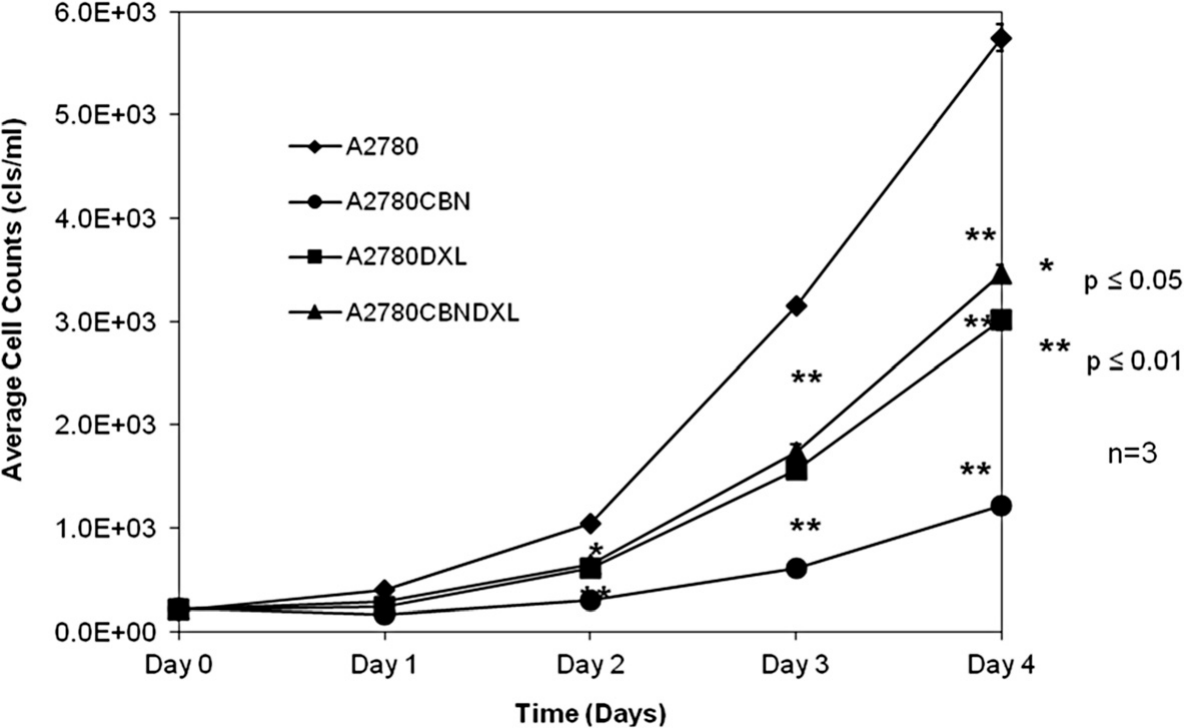
**Proliferation of resistant and parental cell lines.** Average cell counts of three wells, for three replicate experiments, are plotted against time in days. Cell lines were plated at the same density on day 0 and a set of wells was counted on each day of the proliferation assay.

Terminal dose cultures of the A2780CBN, A2780 DXL and A2780CBNDXL cell lines and the parental A2780 line, were plated without drug to determine the effect of selection on cell doubling time (growth rate). Fresh medium was provided to the day 3 and4 cultures on day 2. Curves representing the average proliferation of each cell line across three replicate experiments show that all resistant lines proliferate more slowly than the parental A2780 cell line (Figure 4). Calculation of doubling times for each cell line generated a time of 19.8 hours for the A2780 parental line, 24.02 hours for the A2780CBNDXL line, 25.28 hours for the A2780 DXL line and 39.50 hours for the A2780CBN line, consistent with a reduction in cell doubling time upon selection for resistance to either agent (alone) or the agents in combination.

### Changes in gene expression associated with resistance by microarray analysis

**Figure 5:**
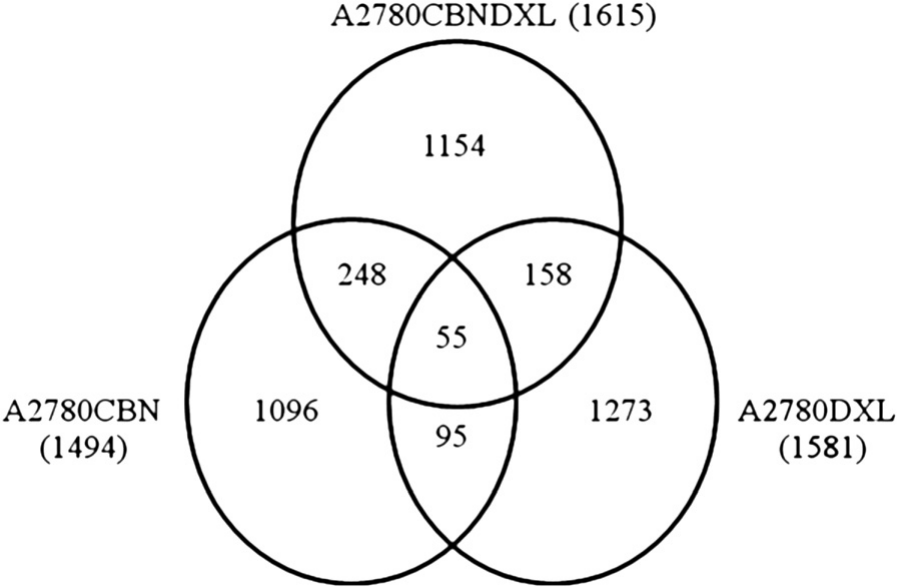
**Unique and shared gene sets among the A2780 resistant cell lines.** The total number of gene expression changes for each line appears in brackets beside the cell line label. The number of changes in gene expression that were unique to each cell line or shared between lines are indicated in appropriate sections.

Lists of genes with significant changes in expression (p ≤ 0.05) in each cell line compared to the matching co-cultured control were derived from the Partek Genomics Suite as described. The Partek Genomics list showed 3000 genes were significantly different in the A2780CBN line, 4621 genes were significant in the A2780DXL line and 4070 genes were significantly different in the A2780CBNDXL line. If a gene exhibited an opposite direction in fold change (upregulated in one line but down regulated in another) it was counted as unique in each line and shared between lines if they went in the same direction. Following the refinement of the lists as described, a total of 1096 unique changes in gene expression were observed for the A2780CBN cell line compared to the co-cultured parental control, 1273 unique changes were observed in the A2780DXL cell line and 1154 changes were specific to the dual resistant cell line (Figure 5). Roughly the same total number of genes were identified as changed as a consequence of selection for drug resistance (irrespective of drug used), but the majority (>70%) of changes in gene expression are unique in each cell line. The number of changes shared between the lines comprised about 15% or less of the total number of changes in each line and the changes shared among all three lines was less than 5%.

**Figure 6:**
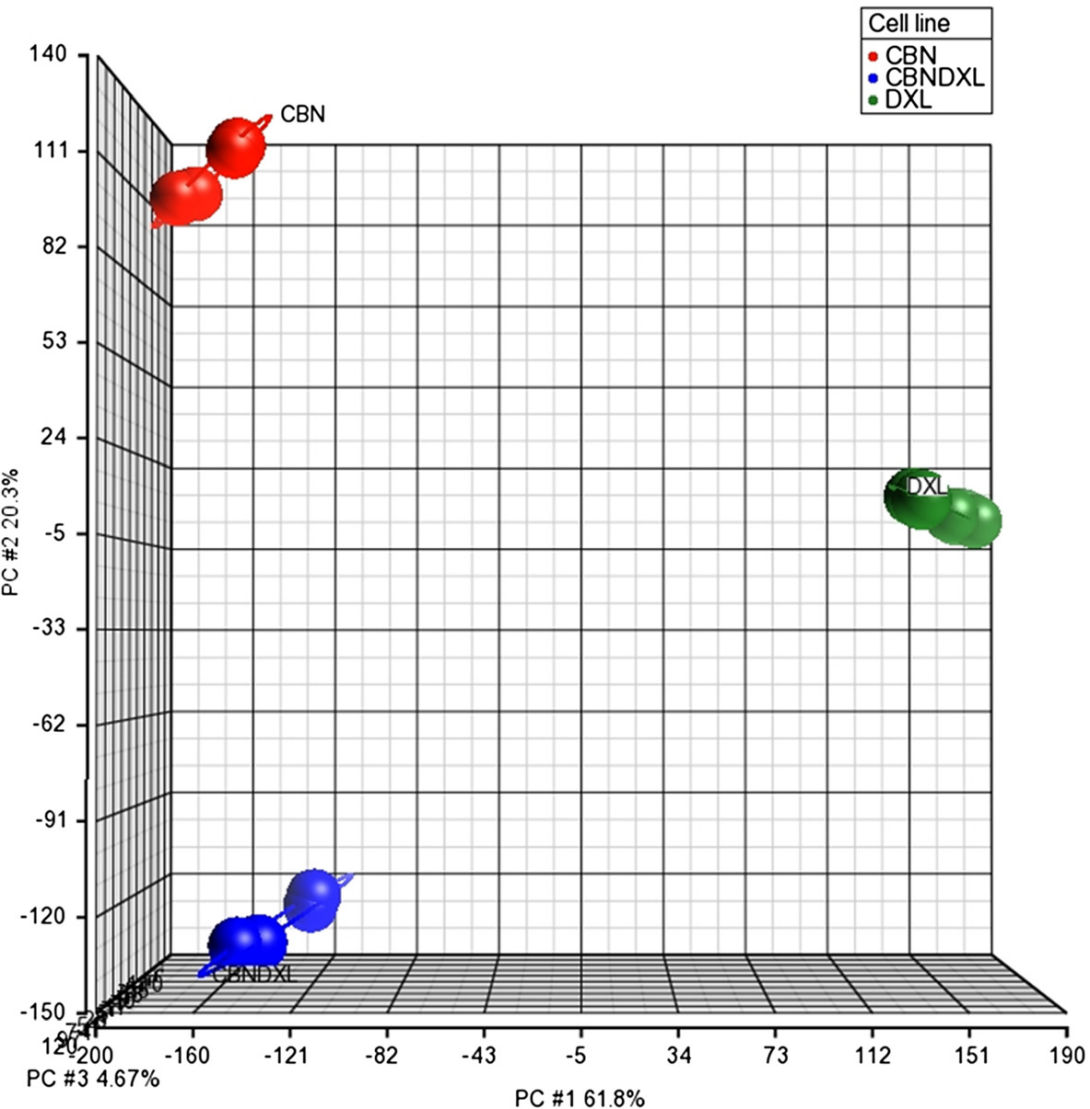
**Principal component analysis of the three resistant A2780 cell lines.** Principal component analysis was performed on the entire set of genes included in the Agilent 4 × 44K whole Human Genome microarray for all the microarray experiments run on the three resistant A2780 cell lines. The analysis was done using the Partek Genomics Suite software.

**Figure 7:**
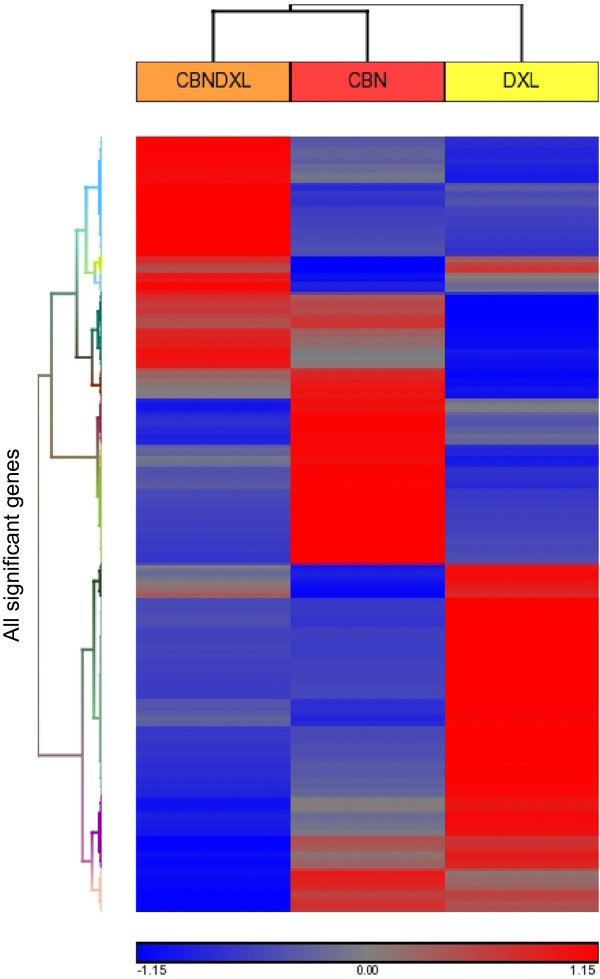
**Hierarchical cluster analysis of gene expression in each resistant cell line.** Heat map showing the result of hierarchical clustering analysis performed in the Partek Genomics Suite software, of all genes from each resistant cell line with significantly different expression compared to the co-cultured control.

Principal Component Analysis of all the genes included in the microarrays was able to separate the gene expression profiles of the samples by drug resistance, indicating that all three of the resistant cell lines were distinct from each other (Figure 6). The first three principal components of the analysis were able to account for about 87% of the total variance in the data, with 62% of the variance accounted for by the first principal component, 20% by the second component and 5% by the third component. The plot in Figure 6 shows that the A2780DXL line is most distinct in terms of gene expression, although the A2780CBN and A2780CBNDXL lines are also clearly separate. Hierarchical clustering of all the genes with significantly altered expression in at least one of the three resistant cell lines showed a difference in the gene expression patterns of each of the three resistant lines, demonstrating again that the dual resistant line is distinct from the single agent resistant lines (Figure 7). Furthermore, the clustering analysis confirmed the greater separation of the docetaxel resistant line.

### Selection of validation gene sets

Unique gene signals based on the microarray results were selected from each of the three resistant cell lines for validation by QPCR and immunoblotting. Genes known to be associated with resistance to either platinum or taxane agents, with a fold change greater than or equal to two, and with average fluorescence signals of 100 units or more were selected. In some cases a gene signal was shared between cell lines; for example the AKR1C3 signal is significantly different in both the A2780CBN and the A2780CBNDXL line. Since the AKR1C3 aldoketoreductase has been shown to play a role in drug resistance [43-45] and the values in the A2780CBNDXL line were within the limits set for the validation gene set, AKR1C3 was included in the gene set despite low values in the A2780CBN line. A similar selection was made for the CDH7 cadherin gene. In the A2780CBNDXL cell line, the CDH7 gene transcript displayed one of the highest fold changes and fluorescence values, while the A2780CBN line displayed a much weaker upregulation. The final validation set selected included a total of 16 genes (Tables 2 and 3), composed of 4–5 genes per line with two genes chosen because the change in expression was shared among all three lines (LAYN and PRSS7) (Table 3).

**Table 2:** **Validation gene set**, **genes with significant changes in one or more of the resistant cell lines according to microarray analysis**

**Cell line**	**Gene designation**	**Protein name**	**General function**	**Fold change**	**P value for fold change**	**Fluorescence value resistant line**	**Fluorescence value parent line**
**A2780CBN**	AKR1C3	Aldo-keto reductase family 1 member C3	Catalyzes the conversion of aldehydes and ketones to alcohols	−3.07	4.94 x 10^-3^	18.75	56.25
	ANXA1	Annexin A1	Calcium dependent phospholipid binding protein	−104.15	1.38 x 10^-4^	96.50	9992.50
	CDH7	Cadherin 7	Cell to cell adhesion glycoprotein	2.08	1.29 x 10^-4^	67.25	32.75
	GCLC	Glutamate-cysteine ligase catalytic subunit	Rate limiting enzyme of glutathione synthesis	8.50	5.85 x 10^-5^	6907.25	815.00
	GSTO1	Glutathione S-transferase omega 1	Stress response protein, catalyzes addition of glutathione to toxic substrates	2.38	1.07 x 10^-3^	23166.50	9726.50
	MT2A	Metallothionein 2A	Heavy metal binding protein	3.69	4.15 x 10^-6^	4899.50	1266.00
	PARP9	Poly(ADP)-ribose polymerase family, member 9	Catalyzes addition of ADP-ribose moieties to substrate proteins	9.40	1.95 x 10^-3^	2523.25	275.25
**A2780DXL**	CYP1B1	Cytochrome P450 family 1, subfamily B, polypeptide 1	Phase 1 enzyme in drug metabolism	−37.77	2.00 x 10^-8^	491.50	14858.38
	LGI1	Leucine-rich glioma inactivated 1	Metastasis regulator	175.85	1.03 x 10^-9^	5449.25	30.00
	ABCB1	ATP-binding cassette transport subfamily B, member 1	Multidrug transporter (MDR/TAP family)	33.62	8.22 x 10^-11^	21065.40	649.60
	ABCB4	ATP-binding cassette transport subfamily B, member 4	Multidrug transporter (MDR/TAP family)	141.27	5.88 x 10^-10^	11876.75	84.63
**A2780CBNDXL**	ABCB1	ATP-binding cassette transport subfamily B, member 1	Multidrug transporter (MDR/TAP family)	35.28	2.43E-03	474.25	13.00
	ABCB4	ATP-binding cassette transport subfamily B, member 4	Multidrug transporter (MDR/TAP family)	22.70	1.89x10^-03^	108.75	5.00
	AKR1C3	Aldo-keto reductase family 1 member C3	Catalyzes the conversion of aldehydes and ketones to alcohols	7.30	2.62 x 10^-3^	400.75	55.75
	FLRT3	Fibronectin Leucine-rich Repeat Transmembrane protein 3	Helps regulate cadherin mediated cell adhesion and cell morphogenesis	−115.40	1.79 x 10^-3^	39.50	4423.25
	GSTO2	Glutathione S-transferase 2	Stress response protein, catalyzes addition of glutathione to toxic substrates	5.34	2.68 x 10^-4^	2133.25	400.00
	CDH7	Cadherin 7	Cell to cell adhesion glycoprotein	68.82	9.24 x 10^-4^	5223.75	75.70
	CDH11	Cadherin 11	Cell to cell adhesion glycoprotein	1022.24	1.57 x 10^-4^	10708.75	10.50

**Table 3:** **Validation gene set**, **genes present in all three cell lines according to microarray analysis**

**Gene designation**	**Protein name**	**General function**	**Cell line**	**Fold change**	**P value for fold change**	**Fluorescence value resistant line**	**Fluorescence value parent line**
**LAYN**	Layilin	Binds hyaluronan, may play a role in cell adhesion and motility	A2780CBN	−154.83	5.63 x 10^-4^	88.25	13106.25
			A2780DXL	−12.95	4.64 x 10^-10^	75.00	948.50
			A2780CBNDXL	−145.93	8.16 x 10^-4^	151.75	14364.25
**PRSS7**	TMPRSS15, Transmembrane protease, serine 15/enterokinase	Membrane bound enterokinase	A2780CBN	164.34	9.35 x 10^-3^	1927.75	15.50
			A2780DXL	84.44	2.24 x 10^-8^	3028.75	37.63
			A2780CBNDXL	123.06	1.79 x 10^-3^	6559.00	60.00

### Significant differences confirmed between parent and resistant cell lines

Significant differences in transcript levels from the microarray data were confirmed by QPCR for all transcripts in at least one cell line except the GSTO1 transcript, which was not found to be significantly different from the parent in any of the cell lines, despite the microarray results (Additional file 2: Table S2). A comparison of fold changes calculated from the microarray and QPCR data is shown in Table 4. There was perfect concordance between the microarray and QPCR results for significant changes in expression of the ABCB1, ABCB4, AKR1C3, GCLC, LAYN and PRSS7 genes. In general, the QPCR experiments confirmed the microarray results with regard to direction of change, but fold change often differed. Furthermore, expression of transcripts was often detected by QPCR in several of the lines, although our filtered microarray data had indicated that the changes selected for validation were unique to a cell line. Additional significant changes were found in one or more of the resistant lines for the ANXA1, CDH11, CYP1B1, FLRT3, GSTO2, LGI1, MT2A, and PARP9 transcripts. Besides providing confirmation of the microarray results, the QPCR data demonstrate greater sensitivity in detecting gene expression compared to microarray hybridization.

**Table 4:** **Comparison of gene expression fold changes by microarray and Q**-**PCR**

**Gene**	**A2780CBN**		**A2780DXL**		**A2780CBNDXL**	
	**Microarray**	**Q**-**PCR**	**Microarray**	**Q**-**PCR**	**Microarray**	**Q**-**PCR**
ABCB1	NS	NS	33.62	73753.41	35.28	2198.19
ABCB4	NS	NS	141.27	3617.62	22.70	224.52
AKR1C3	−3.07	−6.30	NS	NS	7.30	16.37
ANXA1	−104.15	−154.12	NS	8.53	NS	−6.44
CDH7	5.21	NS	NS	NS	68.82	329.30
CDH11	NS	NS	NS	12.10	1022.24	761.14
CYP1B1	NS	NS	−37.77	−517.88	NS	3.57
FLRT3	NS	−342.64	NS	−933.12	−115.40	−101.97
GCLC	11.45	9.98	NS	NS	NS	NS
GSTO1	2.38	NS	NS	NS	NS	NS
GSTO2	NS	NS	NS	9.13	5.34	10.06
LAYN	−154.83	−7275.97	−12.95	−12945.21	−11.03	−1214.05
LGI1	NS	−0.64	175.85	143.67	NS	NS
MT2A	3.87	NS	NS	2.86	NS	NS
PARP9	9.40	2.10	NS	2.95	NS	18.46
PRSS7	164.34	160.48	84.44	487.41	123.06	802.50

NS = not significantly different between parent and resistant line.

### The dual resistant line contains specific differences in gene expression

To examine if the expression level of the selected genes was significantly different between the resistant cell lines, one-way ANOVA was performed on the log of the fold change as determined by QPCR. When the ANOVA showed a significant difference (p ≤ 0.05), Tukey’s test was applied as the post hoc test to identify the cell line(s) that contained most of the difference. Significant differences among the resistant cell lines were not found for five genes (FLRT3, GSTO1, LAYN, MT2A, PRSS7) while two genes (ABCB1 and ANXA1) were significantly different among all three cell lines (Table 5). Of the remaining nine genes in the validation set, four were found to be significantly different in the dual resistant line (AKR1C3, CDH7, CDH11, PARP9) while the A2780CBN and A2780DXL lines each contained only two of the significantly different genes. Differences in expression of the ABCB4 gene could not be assessed among the three resistant cell lines because there was no detectable expression at all in the A2780CBN line. Table 5 lists the pair wise comparison results from the Tukey’s post hoc test and indicates whether a significant difference (p < 0.05, n=3) exists for each comparison. Plots of the log fold changes for the validation gene set showing the results of the ANOVA followed by Tukey’s test are shown in Additional file 3: Figure S1. The results demonstrate that the A2780CBNDXL cell line contains specific changes associated with development of dual drug resistance which are significantly different from the single agent resistant cell lines.

**Table 5:** **Tukey**’**s post hoc test for significant difference among the resistant cell lines**

**Gene designation**	**F**-**test(p Value)**	**A2780CBN vs A2780DXL**	**A2780CBN vs A2780CBNDXL**	**A2780DXL vs A2780CBNDXL**
		**p < 0.05**	**P < 0.05**	**P < 0.05**
ABCB1	2.23E-05	Yes	Yes	Yes
ANXA1	1.19E-04	Yes	Yes	Yes
FLRT3	7.57E-02	No	No	No
GSTO1	3.44E-01	No	No	No
LAYN	4.76E-02	No	No	No
MT2A	2.78E-01	No	No	No
PRSS7	2.00E-01	No	No	No
AKR1C3	3.70E-03	No	Yes	Yes
CDH7	1.88E-02	No	Yes	Yes
CDH11	3.47E-04	No	Yes	Yes
PARP9	9.73E-03	No	Yes	Yes
GCLC	7.27E-05	Yes	Yes	No
GSTO2	2.18E-03	Yes	Yes	No
CYP1B1	3.82E-05	Yes	No	Yes
LGI1	9.61E-06	Yes	No	Yes
ABCB4	*1.92E-02	N.A.	N.A.	*N.A.

*t-test p=0.019.

### Changes in protein expression determined by immunoblotting

**Figure 8:**
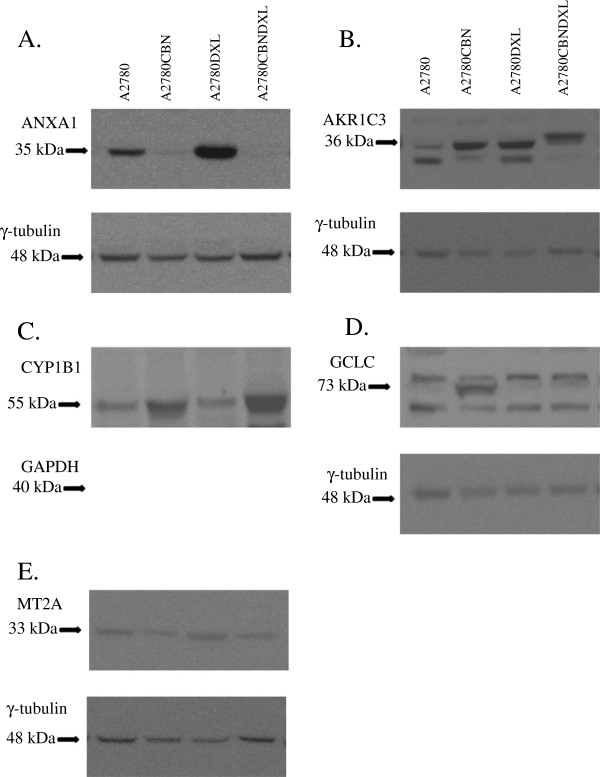
**Immunoblots demonstrating protein expression in the A2780 and resistant cell lines.** Representative immunoblots of protein expression in the A2780, A2780CBN, A2780DXL and A2780CBNDXL cell lines are shown in panel: **A**. Annexin 1 (ANXA1), **B**. Aldoketoreductase family 1 member C3 (AKR1C3), **C**. Cytochrome P450 family 1, subfamily B (CYP1B1), **D**. Glutamate-cysteine ligase catalytic subunit (GCLC), **E**. Metallothionein 2A (MT2A). A loading control immunoblot for either γ-tubulin or glyceraldehyde phosphate dehydrogenase (GAPDH) is shown below each panel.

**Figure 9:**
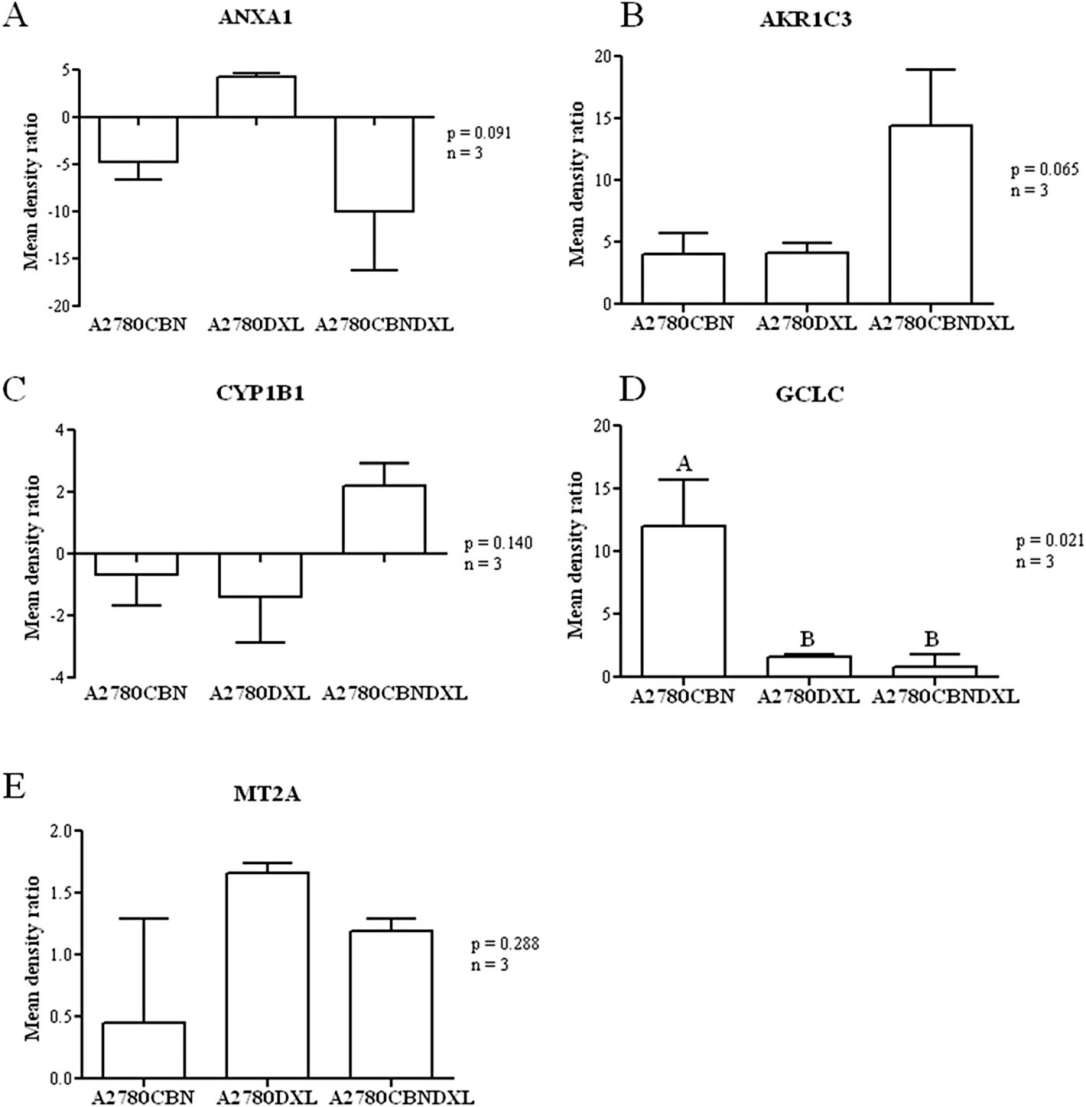
**Comparison of changes in protein expression detected by immunoblotting.** Normalized band densities of immunoblots (n = 3) were used to calculate ratios between the parental and resistant lines which were considered to represent fold change in protein expression between the parental and resistant lines. The fold changes were compared by one-way ANOVA followed by Tukey’s test to determine if there was any significant difference among the cell lines in the expression of each protein and if the difference could be assigned to any cell line(s). Significant difference between cell lines is indicated by lettering above the columns.

Further confirmation of the changes in gene expression, at the protein level, was attempted by immunoblotting. Of the 11 antibodies acquired for the immunoblotting (see Materials and Methods) experiments, only the AKR1C3, ANXA1, CYP1B1, GCLC, GAPDH, MT2A and γ-tubulin antibodies produced measurable immunoblot signals (Figure 8). Following blotting, detection of the primary antibody signal, and stripping of the membrane, a loading control blotting was performed with either the γ-tubulin or GAPDH antibody. After normalization to the loading control band densities, average band density ratios were calculated between the resistant lines and the A2780 parent line (Figure 9). A one way ANOVA followed by Tukey’s post hoc test was calculated for the density ratios and the resulting p values are shown in Figure 9. Of the five successful experiments, only the GCLC results (Figure 9D) indicated that there was a significant difference among the resistant cell lines with most of the difference occurring in the A2780CBN cell line, although a trend towards significantly different expression of AKR1C3 in the dual resistant line is observable.

## Discussion

### Selection of resistant cell lines

In this study, a set of three isogenic drug-resistant ovarian cancer cell lines has been generated from the A2780 ovarian cancer cell line. The A2780 cell line has the advantage of being derived from a chemo-naïve patient, and is therefore sensitive to many chemotherapeutics [46-50] but has been demonstrated to be capable of developing resistance *in vitro*[51-55]. In addition, the strategy of deriving isogenic drug resistant cell lines from one cell line eliminates variability due to intrinsic genetic differences between cell lines. Although numerous studies have investigated mechanisms of drug resistance to single agents, the standard of care for chemotherapy in ovarian cancer is a combined treatment with a platinating agent and a taxane. Therefore, in this study, we report the generation of dual drug resistance *in vitro* and characterization of cells selected for resistance to both classes of agents.

### Characterization of levels of resistance

During the selection for single or dual drug resistance in our study, the gradual increase in drug concentration, beginning with a dose 1000-fold below the IC_50_ of the parental A2780 cell line, generated populations of resistant cells and avoided selection of a few drug resistant clones. This selection strategy may not seem to reflect the typical clinical approach of treating patients with high doses delivered in several cycles, but the dose administered to a patient is not likely reflective of the amount of drug that actually reaches a tumor. Studies of intratumoral drug distribution have shown that drug concentrations vary within a tumor, that not all tumor cells may experience a lethal dose, and that other factors such as intratumoral cell heterogeneity and tumor microenvironment interactions can interfere with consistent, high dose delivery of a drug in a tumor [56-58]. Although this situation is very difficult to imitate under *in vitro* conditions, we believe our approach beginning with a low concentration and gradually increasing the dose is more likely to mimic the variable and gradually increasing drug environment in a tumor and to select for a population of drug resistant cells representative of the cell heterogeneity present in tumors. Using this selection method, the A2780CBN cell line was acquired with an IC_50_ of 7.77 × 10^-5^ M carboplatin (Table 1), a concentration similar to the maximally tolerated plasma concentration of carboplatin (3.8 × 10^-5^ M) [59], indicating the A2780CBN cell line tolerates clinically detectable concentrations of carboplatin. The level of resistance in the A2780CBN line (13.56 fold) is comparable to resistance levels reported for cisplatin *in vitro* in ovarian tumor cells [10,52].

The A2780DXL cell line had an IC_50_ of 3.61 × 10^-7^ M docetaxel, which was 4000 fold more resistant than the A2780CC. Although initially very toxic, once resistance had begun to develop, it was possible to increase the dose until this very high level of resistance occurred. Intraperitoneal delivery of docetaxel to patients was reported by Morgan et al. to result in mean peak plasma concentrations of 4.6-6.6 × 10^-7^ M docetaxel and 5.9-8.1 × 10^-5^ M mean peak intraperitoneal concentrations of docetaxel [60]. Although the range between the plasma and intraperitoneal concentrations reported by Morgan et al. is more than 100-fold, depending on the compartment measured, the IC_50_ of our A2780DXL line falls just under the lower end of the range, indicating that the A2780DXL cell line tolerance also falls in a clinically relevant range. *In vitro* resistance to paclitaxel in ovarian cell lines has been reported in this range, as well [61,62].

The selection of the dual resistant A2780CBNDXL cell line resulted in combined resistance, with an IC_50_ of 8.02 × 10^-6^ M for carboplatin and 8.02 × 10^-9^ M docetaxel (Figure 1). Compared to the A2780CC cell line, the fold change in resistance is about 13 for both drugs since the method we used increased the carboplatin and docetaxel doses at the same time and to the same extent. It is interesting that the increase in resistance is about 13-fold which is similar to the A2780CBN line. This may indicate that the carboplatin concentration was the limiting factor in this type of selection scheme. A role for carboplatin in determining the degree of resistance achieved in the A2780CBNDXL lines may be reflected by the principal component and hierarchical clustering analyses which both showed that the A2780CBN and A2780CBNDXL cell lines were more similar to each other than either was to the docetaxel resistant line. If we compare the IC_50_ values for the single agent resistant lines to the dual line (Table 1), there is about a 10-fold decrease in the amount of carboplatin tolerated by the dual line compared to the A2780CBN line and about a 45-fold decrease in the amount of docetaxel tolerated by the dual resistant line, indicating that dual drug treatment is effective at lower doses.

To ensure that the A2780CBNDXL cell line truly was resistant to both carboplatin and docetaxel, we exposed the dual line to each drug alone. Figure 2 shows that the A2780CBNDXL line is resistant to carboplatin (Figure 2A) and to docetaxel (Figure 2B), demonstrating that the A2780CBNDXL line is a dual drug resistant cell line. Compared to the A2780CC, the dual line is 10 fold more resistant to carboplatin and 8 fold more resistant to docetaxel. The degree of resistance to each drug appears to be less than when the dual line is exposed to both drugs simultaneously (13 fold), but this is likely due to the A2780CC line tolerating a higher concentration of drug when it is exposed to each drug alone compared to both drugs simultaneously.

Cross resistance to completely different drugs or compounds in cell lines selected for resistance to a specific drug is a recognized phenomenon [53,63,64]. In contrast, cross resistance between platinating agents and taxanes is not very common [65]. In a review of more than 100 models of acquired drug resistance, approximately 70% of cisplatin resistant and paclitaxel resistant cells remained sensitive to paclitaxel and cisplatin, respectively [8]. Since cross resistance could conceivably contribute to a phenotype of dual drug resistance, the sensitivity of the single drug resistant cell lines to the opposite drug was tested. In this study, both the A2780CBN and A2780DXL lines were shown to lack cross resistance to docetaxel and carboplatin, respectively (Figure 3). Interestingly, the A2780DXL cell line showed a trend towards hypersensitivity towards carboplatin, although this was not statistically significant (Figure 3B). Hypersensitivity occurs when a resistant cell line is more sensitive to a drug than the parental cell line it was derived from [8,44], and was observed in almost 30% of the models of acquired drug resistance surveyed by Stordahl et al. While the lack of cross resistance in the single agent resistant A2780 cell lines does not prove that the dual agent resistant line developed without cross resistance, it seems more likely that a genuine dual resistance was generated in the A2780CBNDXL line and not just a single agent resistance with cross resistance to the opposite drug.

### Proliferation of resistant cell lines

The rates of proliferation determined for each of the resistant cells lines and the co-cultured control line show that all the resistant lines have a reduced rate of proliferation compared to the A2780 parental line (Figure 4). While it is well known that malignant cells exhibit a higher rate of proliferation than normal cells [66-68], it is not as well-established that drug resistant cells may also demonstrate an altered rate of proliferation. Gene expression leading to increased cell proliferation and drug resistance has been reported [69,70]. However, reports of reduced cell proliferation associated with increased drug resistance have also been made and an association between multi-drug resistance and decreased proliferation exists, which supports our observation of decreased proliferation in not only the single agent resistant but the dual agent resistant cell line [13,71,72]. Moreover, reduced proliferation in drug resistance may not be so surprising when one considers that most cytotoxic chemotherapy agents are designed to target rapidly proliferating cells; reduction of proliferation could be one way to promote a drug resistant phenotype.

### Microarray analysis of gene expression patterns in the resistant A2780 cell lines

The number of unique changes in gene expression detected in each cell line was similar (Figure 5). Considering the different mechanisms of action of carboplatin and docetaxel, it is expected that the carboplatin and docetaxel resistant cell lines should not have many changes in gene expression in common. However, the relatively low amount of common gene expression changes between the dual line and each of the single agent resistant lines indicates that the majority of the changes in the dual line are unique and not a simple combination of the patterns present in each single agent resistant line. Furthermore, the separation of the three resistant cell lines by principal component analysis of all the genes with altered expression supports our claim of a distinct pattern of gene expression in the dual resistant cell (Figure 6). Additional evidence for the unique pattern of gene expression induced by simultaneous exposure of the cells to both carboplatin and docetaxel is present in the hierarchical cluster analysis which shows a different pattern of gene expression in all three resistant cell lines (Figure 7). Based on these results, we can state that development of resistance to more than one chemotherapy agent has the potential to induce novel changes not associated with resistance to each single agent.

### Validation of microarray results

QPCR amplification of validation gene set transcripts confirmed the results of the microarray analysis, except for the GSTO1 gene, which was not confirmed by QPCR as significantly upregulated in the A2780CBN line, although expression was detected by microarray hybridization (Table 4, Additional file 2: Table S2). The QPCR results were more sensitive in detecting changes in gene expression not found by microarray analysis. For example, 11 additional instances of altered gene expression were detected by QPCR for ANXA1, CDH11, CDH7, CYP1B1, FLRT3, GSTO2, LGI1, MT2A, and PARP9 (Table 4). Fold changes were in the same direction but the QPCR results often showed a much greater change, e.g. the ABCB1 and ABCB4 gene expression detected by QPCR was around 10–1000 greater than the microarray results (Table 4). The improved accuracy of detecting gene expression by QPCR in our study may be due to the design of the QPCR primers, which were based on transcript specific sequences from the protein coding transcript for each gene whereas the oligonucleotides used in the microarray are designed to detect all possible transcripts of a gene, including non-coding transcripts. Therefore, our QPCR primers are more accurate in detecting gene expression that is more likely to be associated with protein expression and represent true genetic response to drug selection.

### QPCR confirmation of differences in gene expression among the three resistant A2780 cell lines

The one way ANOVA followed by Tukey’s post hoc test detected significant differences in expression among the resistant cell lines as determined by QPCR. Based on this analysis, four of the genes in the validation set of 16 genes, were found to be significantly different in the A2780CBNDXL line. Although also significant in the A2780CBN line, the AKR1C3 gene was expressed to a significantly different extent mainly in the dual resistant line. The role of aldoketoreductases in cisplatin and multidrug resistance has been described in several different types of cancer cells [43,45,73,74]. Therefore, the discovery of a significant increase in AKR1C3 expression in the dual drug resistant line supports a role for aldoketoreductases in combined carboplatin and docetaxel resistance. The PARP9 gene was also mainly expressed in the dual drug resistant line. PARP proteins, in particular PARP 1, are involved in DNA repair and have become a therapeutic target in BRCA mutant cancers [75-77]. A direct role for PARP proteins has also been reported in cisplatin resistance [78,79]. In this study we report a significant increase in expression of PARP9 in the dual resistant A2780CBNDXL line compared to the A2780CBN line, extending the impact of PARP proteins to combined carboplatin and docetaxel resistance. An additional two genes that were mainly expressed in the dual line were CDH11 and CDH7, with CDH11 being the most upregulated gene in the dual line (1022 fold upregulated). Cadherins, in particular CDH1 (E-cadherin), are known to contribute to invasiveness and stem cell like properties in ovarian cancer [80-83]. E-cadherin-mediated intercellular adhesion has also been shown to contribute to chemotherapy resistance [84]. CDH11, however, is a classic type II cadherin, known to be involved with bone morphogenesis [85], and has been shown to play a role in epithelial to mesenchymal transition [86]. As well, CDH11 mediates cell adhesion [87,88] as does CDH7 [89,90], another classic type II cadherin, also significantly over expressed in the dual line. Intercellular adhesion has been demonstrated as an important factor in multidrug resistance [72]. Therefore, the distinct upregulation of CDH11 and CDH7 in the dual resistant A2780CBDXL cell line could indicate a role for type II cadherin mediated cell adhesion in this type of combined drug resistance.

The A2780CBN cell line contained most of the significant difference for two genes, GCLC and GSTO2. GCLC codes for γ-glutamylcysteine synthetase which controls the rate limiting step in the synthesis of glutathione while GSTO2 produces glutathione S-transferase omega 2. The combination of the two is known to play a role in anticancer drug resistance, including cisplatin resistance [11,91,92]. The increased expression of both genes in the A2780CBN line confirms that the importance of the glutathione pathway in carboplatin resistance, besides cisplatin resistance. A novel change with most of the difference in expression contained in the A2780DXL line is the upregulation of the LGI1 gene, which we have found in another docetaxel resistant line (MCF7txt) (A. Parissenti, unpublished data). The LGI1 gene was originally observed in glioma where increased expression of LGI 1 contributes to decreased proliferation of neuroblastoma cells [93,94]. A decrease in proliferative capacity, as we observed for A2780DXL (Figure 3), could be promoted by changes in genes like LGI1. Overexpression of cytochrome enzymes, especially of the CYP 450 3A family [95,96] are known to play a role in the metabolism of docetaxel. However, the A2780DXL line contained most of the significant difference for another cytochrome, CYP1B1, which does not play a role in metabolism of the drug although increased expression of CYP1B1 has been shown to be associated with resistance to docetaxel [97]. However, an oxidized CYP1B1 estrogen metabolite has been reported to inhibit tubulin polymerization [98]. Interestingly, expression levels of CYP1B1 are down regulated in our A2780DXL line (Table 4, Additional file 3: Figure S1), which contradicts the study by Martinez et al. [97], but seems to support the role of docetaxel in inhibiting tubulin polymerization reported by Sissung *et al*. [98].

Other genes found to be significantly different in the resistant lines compared to the parent line, were not mainly expressed in any one of the cell lines. The ABCB1 and ANXA1 genes, although previously shown to be associated with drug resistance [10,14] were significantly different in all three cell lines, showing major changes in expression, but without any one line containing most of the difference. The remaining genes (Table 5) displayed a very similar change in expression across the cell lines without significant distribution of expression to one cell line.

### Immunoblot confirmation of changes in protein expression

Immunoblots were performed to determine if changes in gene expression at the transcript level could be confirmed at the protein expression level. Of the five successful immunoblots, only the GCLC protein demonstrated a significantly different degree of expression in a cell line; upregulation in the A2780CBN cell line (Figures 8 and 9), confirming the glutathione pathway as a strong component of the resistance mechanisms in the A2780CBN cell line. However, the immunoblot data confirm the ANOVA results for both the ANXA1 and MT2A protein expression. As shown in Table 5, all three resistant cell lines display variable and quite different expression of ANXA1 transcripts and this is reflected by the immunoblot results (Figures 8 and 9). Although the MT2A blots seem to show a noticeable difference in the A2780DXL line, the fluctuating amounts of protein detected support the conclusion of no significant difference among cell lines displayed in Table 5. It is curious that both the ANXA1 and MT2A blots contradict the expectation from the microarray data, which indicated that expression of these two genes was specific to the carboplatin resistant line. The CYP1B1 blot follows the same trend of not supporting A2780DXL specific down regulation although this was demonstrated by both microarray and QPCR analysis. Finally, despite lack of statistical significance, expression of the AKR1C3 protein tends to be greatest in the dual resistant A2780CBNDXL line, which would support the microarray and QPCR results demonstrating a significant association of this gene with combined carboplatin and docetaxel resistance. The low concordance between the microarray, Q-PCR and protein expression data is not entirely surprising as this has been observed in other studies of gene and protein expression [99-101]. These studies show that there is not always a direct correlation between transcription levels and translation of a gene product, which indicates that caution should be observed in assuming that gene expression data can predict protein levels. Accurate knowledge of gene translation requires assessment of protein expression.

## Conclusions

In this study, we report the establishment of a novel cell line with documented resistance to both carboplatin and docetaxel. Microarray analysis and QPCR confirmation of changes in expression of selected genes show that the dual resistant cell line contains specific genetic alterations not present in either carboplatin or docetaxel resistant cell lines which were selected in an identical manner in the same study using the same source of A2780 cells. These results demonstrate that combined drug resistance is not just a simple combination of changes present in single agent resistant cells but can contain novel and different changes. The dual carboplatin-docetaxel resistant cell line will facilitate further investigation into mechanisms underlying the development of dual drug resistance in ovarian cancer.

## Abbreviations

RIN: RNA integrity; FBS: Fetal bovine serum; PCR: Polymerase chain reaction; QPCR: Quantitative Real-Time Polymerase Chain Reaction; IMDM: Iscove’s Modified Dulbecco’s Medium; RPMI: Roswell Park Modified IMDM; A2780CC: Co-cultured parental control cells; A2780CBN: A2780 cell selected for resistance to carboplatin; A2780DXL: A2780 cells selected for resistance to docetaxel; A2780CBNDXL: A2780 cells selected for resistance to carboplatin and docetaxel; IC_50_: Inhibitory concentration at which 50% survival occurs; Ct: Cycle threshold; the number of PCR cycles at which the product signal becomes detectable (exceeds background value).

## Competing interests

The authors of this paper have no competing interests to declare. We have not received reimbursements, fees, funding, or salary from an organization that may in any way gain or lose financially from the publication of this manuscript, either now or in the future. No such organization is financing this manuscript, including the article-processing charge. We do not hold stocks or shares in any organization that may gain or lose financially from the publication of this manuscript, either now or in the future. We do not hold and are not currently applying for any patents relating to the content of the manuscript. We have not received reimbursements, fees, funding, or salary from an organization that holds or has applied for patents relating to the content of the manuscript. We have no other financial competing interests. We have no non-financial competing interests (political, personal, religious, ideological, academic, intellectual, or commercial) to declare in relation to this manuscript. The author(s) declare that they have no competing interests.

## Authors’ contributions

SRA generated the resistant cell lines, assisted in performing the microarray experiments, and performed the data subset analysis and selection of the validation genes. RN performed and analyzed the qPCR validation experiments for the selected genes. BG carried out the microarray experiments and the initial data analysis to identify genes with significantly different expression in the resistant cell lines. AP contributed to the conception of the study, reviewed the manuscript and oversaw the microarray experiments. KLM performed the proliferation analysis of the cell lines and analyzed the data. SC carried out the immunoblots and generated the graphs. CL contributed to the conception of the study, oversaw the selection of the cell lines, oversaw the proliferation analysis, contributed to the final data analysis and drafted the manuscript. All authors read and approved the final manuscript.

## Supplementary Material

Additional file 1 **Table S1.** Primer sequences and melting temperatures. Click here for file

Additional file 2 **Table S2.** Determination of significant difference between the parent and resistant cell lines according to QPCR analysis. Click here for file

Additional file 3 **Figure S1.** Comparison of gene expression changes between the resistant cell lines. The log fold change in gene expression is shown for each resistant cell line. One-way ANOVA followed by Tukey’s test was performed to determine if there was any significant difference among the cell lines in the level of gene expression and if the difference could be assigned to any cell line(s). Significant difference between cell lines is indicated by lettering above the columns. Click here for file
