# Supplementary material for: Distinct genetic alterations occur in ovarian tumor cells selected for combined resistance to carboplatin and docetaxel
Source: J Ovarian Res. 2012 Nov 30;5:40. doi: 10.1186/1757-2215-5-40 (PMC3541348; doi:10.1186/1757-2215-5-40)
Supplement: Additional file 1 — Table S1. Primer sequences and melting temperatures. [file 1757-2215-5-40-S1.doc]

Supplemental Table 1. Primer sequences and melting temperatures

| **Name** | **Sequence (5’-3’)** | **Melting Temperature (°C)** |
| --- | --- | --- |
| *ABCB1 - Forward | GCAGCTGGAAGACAAATACACAA | 55.8 |
| *ABCB1 - Reverse | CCCAACATCGTGCACATCA | 55.8 |
| *AKR1C3 - Forward | GAAGTAAAGCTTTGGAGGTCA | 52.5 |
| *AKR1C3 - Reverse | GTCAACATAGTCCAATTGAGC | 51.5 |
| ABCB4 - Forward | TGGTATGGATCCACTCTAGTCA | 54.4 |
| ABCB4 - Reverse | GTTAGCTCGAGAAGGGTAAGAA | 53.8 |
| ANXA1 - Forward | TCTGGCCAAAGACATAACCT | 53.7 |
| ANXA1 - Reverse | GTATTTCTGAAACACTCTGCGA | 52.9 |
| CDH11 - Forward | TCATCAGAACAGCCCTACCC | 56.4 |
| CDH11 - Reverse | TGAGAGTCCGCCCATATGTC | 56.4 |
| CDH7 - Forward | TGGTCTACAGTATTCTGCAAGG | 54.2 |
| CDH7 - Reverse | CAACTGAGGCTACAGGTAATGA | 54.2 |
| CYP1B1 - Forward | GTGGAAGGCAGAGAAATCTAAG | 53.1 |
| CYP1B1 - Reverse | AGTTGTAATGGGAGTTCTGTCC | 54.4 |
| FLRT3 - Forward | GCAATACCCAACACAGTGTATC | 53.8 |
| FLRT3 - Reverse | AGGTAGAGCAAGTTTCCAAGAG | 54.4 |
| GCLC - Forward | AGACTACCTGGGACCTGATTTA | 54.7 |
| GCLC - Reverse | GTGCTAGAAACCCAAGATTACC | 53.3 |
| GSTO1 - Forward | CAAGCTAGAGGAGGTTCTGACT | 55.7 |
| GSTO1 - Reverse | TCAGTTTTGGAGTGTGGTCTAC | 54.5 |
| GSTO2 - Forward | TGAGTATCAGAACACCACCTTC | 54.1 |
| GSTO2 - Reverse | CACAGTCCAGTATCCCATACAC | 54.7 |
| LAYN - Forward | TAGGAACTGGTATGTGGATGAG | 53.5 |
| LAYN - Reverse | AGATTCAAGGCAGCTTCTCTAC | 54.4 |
| LGI1 - Forward | GACAACATTACAGGCACATCC | 53.9 |
| LGI1 - Reverse | TAGAATCCGTTTCCGTTCCA | 53.6 |
| MT2A - Forward | TGCAACCTGTCCCGACT | 56.6 |
| MT2A - Reverse | TCACGGTCAGGGTTGTACAT | 55.9 |
| PARP9 - Forward | CTCCATCACAGAAATTATCAGCC | 53.6 |
| PARP9 - Reverse | AATAGTCCACTGTCCTAACGAG | 53.6 |
| PRSS7 - Forward | CTAGACAAGCTAACAACCACCA | 54.6 |
| PRSS7 - Reverse | GGATAATGAGTAGCCTGGAAAG | 52.7 |
| S28 - Forward | TCCATCATCCGCAATGTAAAAG | 53.4 |
| S28 - Reverse | GCTTCTCGCTCTGACTCCAAA | 57.1 |

Primers were designed using PerlPrimer v1.1.19 software (copyright 2003-2010 Owen Marshall), OligoPerfect^TM^ Designer (Invitrogen, Carlsbad, CA, USA) and NCBI Primer Blast (NCBI, NLM, NIH, Bethesda, MD, USA) and synthesized by Integrated DNA Technologies, Inc. (Windsor, Ontario, CA). Primers used to amplify the ABCB1 transcript were verified in a study by Hembruff *et al*., 2008, BMC Cancer 8:318. Primers used to amplify the AKR1C3 transcripts were verified in a study by Veitch et al., 2009, Pharmacogenet. Genomics 19(6):477.
