# Supplementary material for: Distinct genetic alterations occur in ovarian tumor cells selected for combined resistance to carboplatin and docetaxel
Source: J Ovarian Res. 2012 Nov 30;5:40. doi: 10.1186/1757-2215-5-40 (PMC3541348; doi:10.1186/1757-2215-5-40)
Supplement: Additional file 2 — Table S2. Determination of significant difference between the parent and resistant cell lines according to QPCR analysis. [file 1757-2215-5-40-S2.doc]

Supplemental Table 2. Determination of significant difference between the parent and resistant cell lines according to QPCR analysis

| **Gene** | **A2780 CBN** | | | **A2780 DXL** | | | **A2780 CBNDXL** | | |
| --- | --- | --- | --- | --- | --- | --- | --- | --- | --- |
|  | Normalized average log Ct parent | Normalized average log Ct resistant | p-value | Normalized average log Ct parent | Normalized average log Ct resistant | p-value | Normalized average log Ct parent | Normalized average log Ct resistant | p-value |
| ABCB1 | 1.28 | 1.26 | 0.17 | 1.28 | 1.04 | 1.98E-05 | 1.28 | 1.14 | 9.91E-04 |
| ABCB4 | 1.25 | - | - | 1.25 | 1.09 | 0.03 | 1.25 | 1.14 | 0.03 |
| AKR1C3 | 1.16 | 1.18 | 3.91E-02 | 1.16 | 1.18 | 6.39E-02 | 1.16 | 1.12 | 1.58E-02 |
| ANXA1 | 1.06 | 1.17 | 3.36E-04 | 1.06 | 1.01 | 6.60E-03 | 1.06 | 1.10 | 1.91E-02 |
| CDH11 | 1.24 | 1.23 | 0.83 | 1.24 | 1.18 | 1.57E-02 | 1.24 | 1.09 | 6.73E-04 |
| CDH7 | 1.17 | 1.17 | 0.91 | 1.17 | 1.18 | 0.69 | 1.17 | 1.08 | 2.58E-03 |
| CYP1B1 | 1.07 | 1.08 | 0.25 | 1.07 | 1.19 | 2.65E-04 | 1.07 | 1.05 | 1.83E-02 |
| FLRT3 | 1.09 | 1.20 | 1.99E-04 | 1.09 | 1.21 | 1.67E-03 | 1.09 | 1.15 | 2.37E-02 |
| GCLC | 1.10 | 1.05 | 4.58E-05 | 1.10 | 1.10 | 0.09 | 1.10 | 1.10 | 0.90 |
| GSTO1 | 1.08 | 1.07 | 0.23 | 1.08 | 1.08 | 0.98 | 1.08 | 1.08 | 0.68 |
| GSTO2 | 1.14 | 1.15 | 0.29 | 1.14 | 1.09 | 3.63E-03 | 1.14 | 1.09 | 2.91E-03 |
| LAYN | 1.07 | 1.24 | 2.52E-04 | 1.07 | 1.24 | 7.84E-05 | 1.07 | 1.21 | 2.18E-04 |
| LGI1 | 1.23 | 1.25 | 1.41E-02 | 1.23 | 1.12 | 2.62E-05 | 1.23 | 1.23 | 0.77 |
| MT2A | 1.13 | 1.11 | 0.25 | 1.13 | 1.10 | 0.04 | 1.13 | 1.09 | 0.15 |
| PARP9 | 1.14 | 1.13 | 3.67E-02 | 1.14 | 1.12 | 1.44E-02 | 1.14 | 1.08 | 4.63E-03 |
| PRSS7 | 1.20 | 1.12 | 9.04E-04 | 1.20 | 1.08 | 8.94E-05 | 1.20 | 1.07 | 1.47E-04 |
