# Supplementary material for: Distinct genetic alterations occur in ovarian tumor cells selected for combined resistance to carboplatin and docetaxel
Source: J Ovarian Res. 2012 Nov 30;5:40. doi: 10.1186/1757-2215-5-40 (PMC3541348; doi:10.1186/1757-2215-5-40)

Supplemental Figure 1. Comparison of gene expression changes between the resistant cell lines.

A. B.


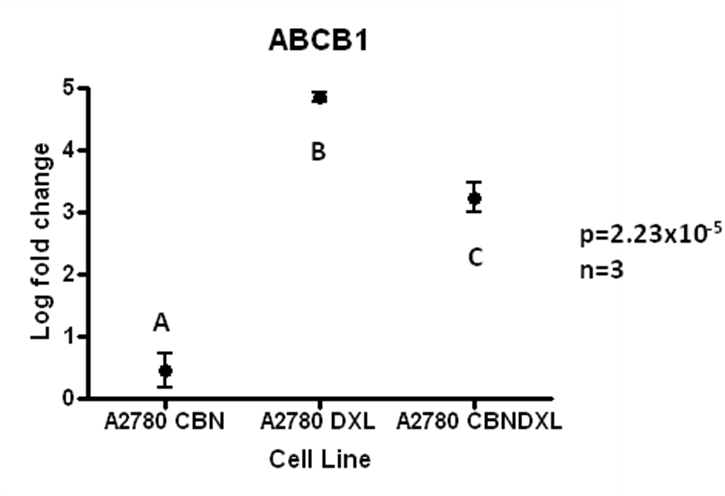

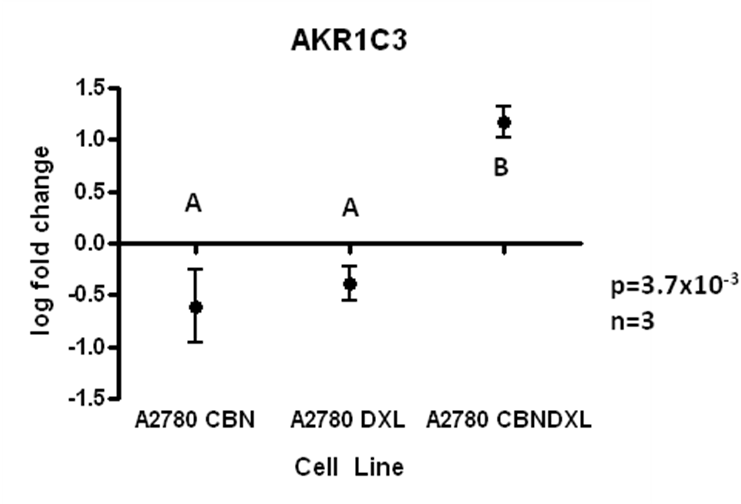


C. D.


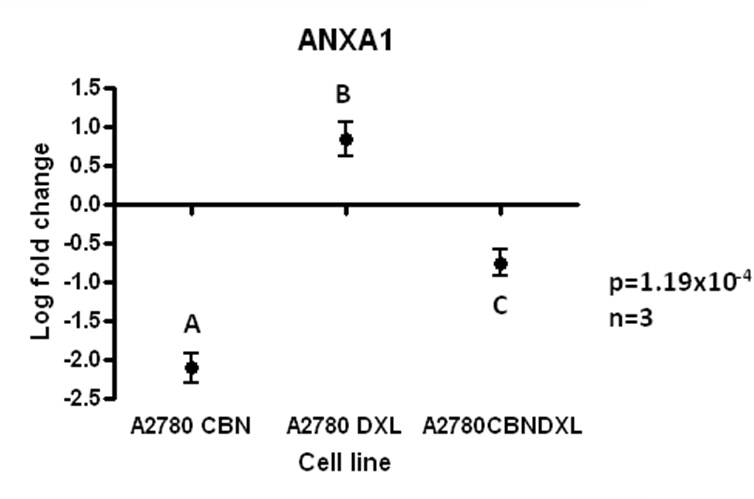

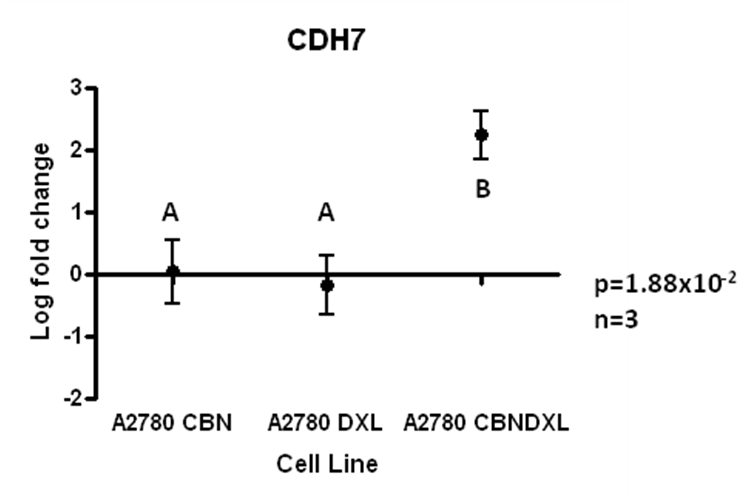


E. F.


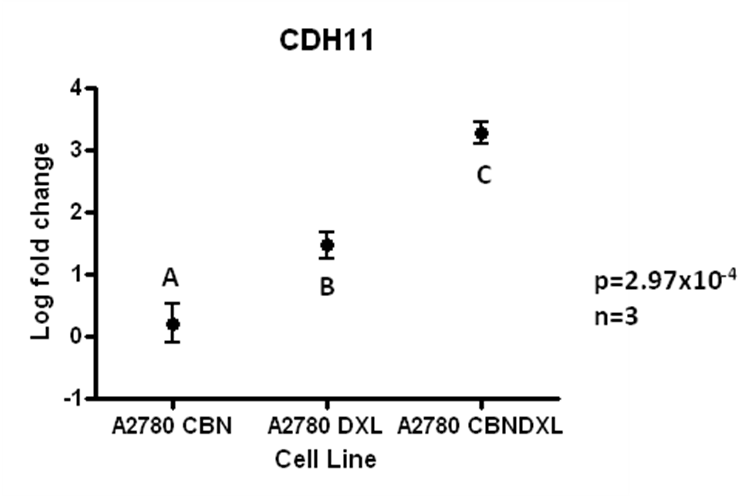

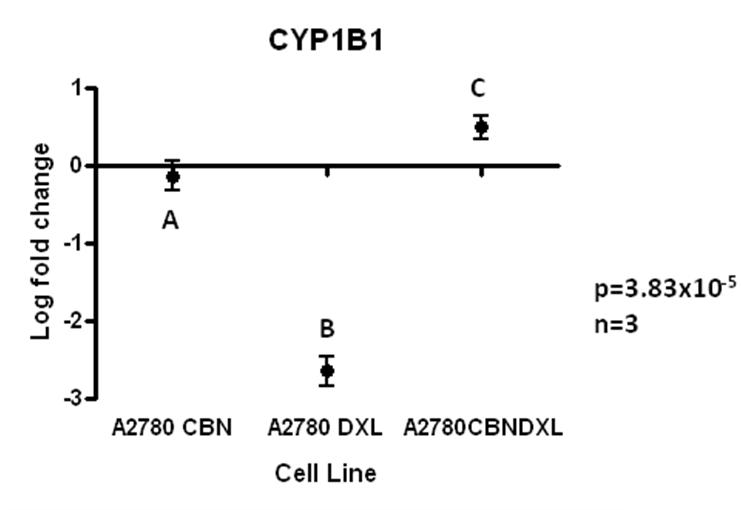


Figure 1(cont.). Comparison of gene expression changes between the resistant cell lines.

G. H.

I. J.


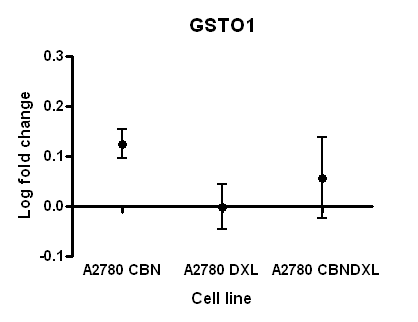

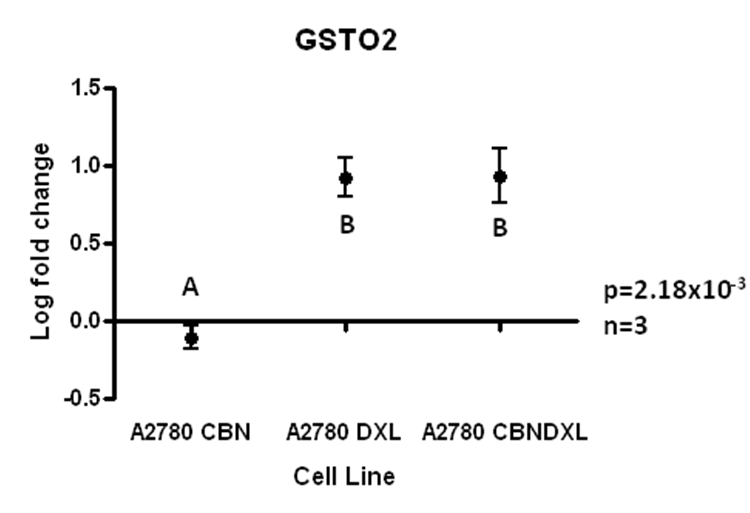


K. L.


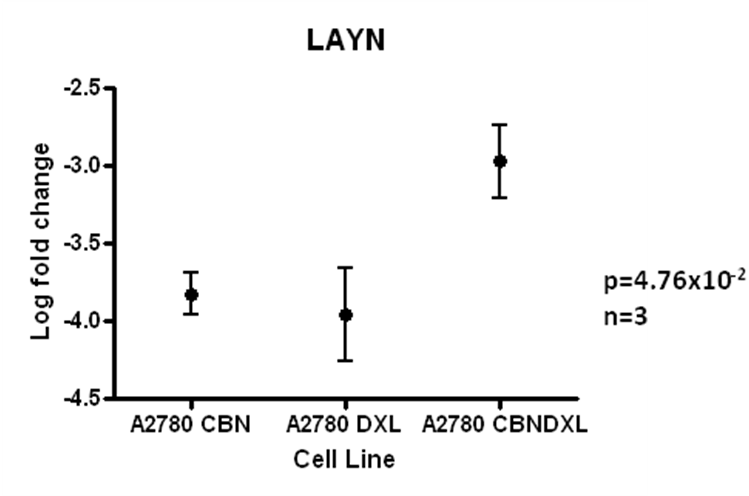

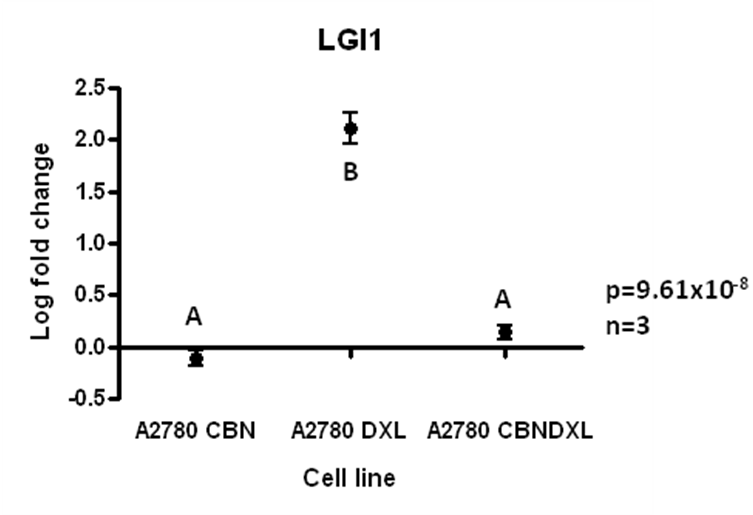


Figure 1(cont.). Comparison of gene expression changes between the resistant cell lines.

M. N.


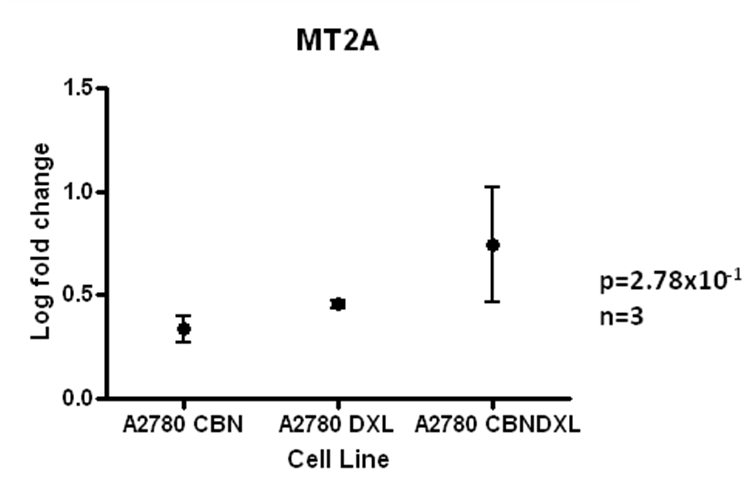

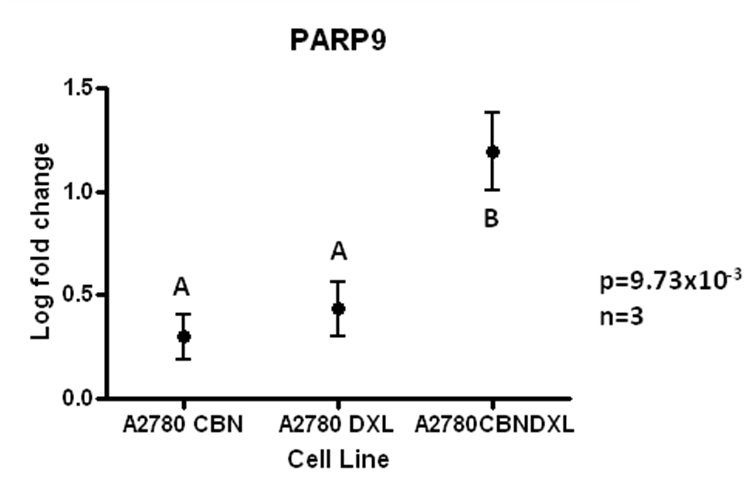


O.


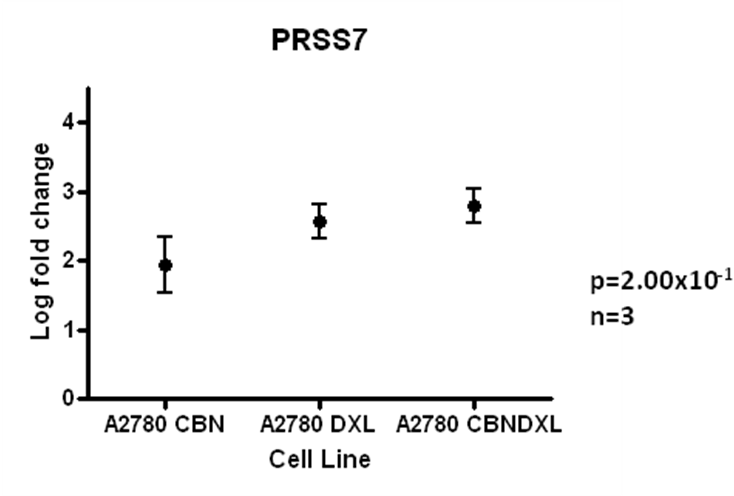

Supplement: Additional file 3 — Figure S1. Comparison of gene expression changes between the resistant cell lines. The log fold change in gene expression is shown for each resistant cell line. One-way ANOVA followed by Tukey’s test was performed to determine if there was any significant difference among the cell lines in the level of gene expression and if the difference could be assigned to any cell line(s). Significant difference between cell lines is indicated by lettering above the columns. [file 1757-2215-5-40-S3.docx]
